# Protective Efficacy of a Candidate Live-Attenuated Vaccine Derived from the SD-R Strain against NADC34-like Porcine Reproductive and Respiratory Syndrome Virus

**DOI:** 10.3390/vaccines11081349

**Published:** 2023-08-09

**Authors:** Hu Xu, Chao Li, Bangjun Gong, Wansheng Li, Zhenyang Guo, Qi Sun, Jing Zhao, Lirun Xiang, Jinhao Li, Yan-Dong Tang, Chaoliang Leng, Qian Wang, Jinmei Peng, Guohui Zhou, Huairan Liu, Tongqing An, Xuehui Cai, Zhi-Jun Tian, Hongliang Zhang

**Affiliations:** 1 State Key Laboratory for Animal Disease Control and Prevention, Harbin Veterinary Research Institute, Chinese Academy of Agricultural Sciences, Harbin 150001, China; xuhu1995@foxmail.com (H.X.); lichao2459@foxmail.com (C.L.); bangjungong@foxmail.com (B.G.); wansheng1994@163.com (W.L.); zhenyang499@163.com (Z.G.); s77arch@163.com (Q.S.); zhaojing94vet@163.com (J.Z.); zhhlxlr@163.com (L.X.); lijinhao990509@163.com (J.L.); tangyandong@caas.cn (Y.-D.T.); wangqian@caas.cn (Q.W.); pjm7614@163.com (J.P.); zhouguohui@caas.cn (G.Z.); liuhuairan@caas.cn (H.L.); antongqing@caas.cn (T.A.); caixuehui139@163.com (X.C.); tianzhijun@caas.cn (Z.-J.T.); 2 Henan Provincial Engineering and Technology Center of Animal Disease Diagnosis and Integrated Control, Nanyang Normal University, Nanyang 473061, China; lenghan1223@126.com

**Keywords:** NADC34-like PRRSV, NADC30-like PRRSV, SD-R, cross-protection

## Abstract

NADC34-like porcine reproductive and respiratory syndrome virus (PRRSV) strains were first detected in China in 2017 and became major circulating strains in 2021. Our previous study showed that the live-attenuated vaccine candidate SD-R strain could provide broad cross-protection against different NADC30-like PRRSVs (sublineage 1.8). However, the protective effect of SD-R against NADC34-like PRRSV is unclear. Here, a novel NADC34-like PRRSV, LNTZJ1341-2012, was isolated from a pig farm experiencing disease in 2020. Sequence analysis revealed that LNTZJ1341-2012 belonged to PRRSV-2 sublineage 1.5, exhibited the same Nsp2 amino-acid deletion characteristics as IA/2014/NADC34, and had not recombined with other strains. Additionally, a good challenge model was established to evaluate the protection afforded by the candidate SD-R vaccine against infection with a representative NADC34-like strain (LNTZJ1341-2012). The control piglets in the challenge experiment displayed clinical signs typical of PRRSV infection, including transient fever, high viremia, mild clinical symptoms, and histopathological changes in the lungs and submaxillary lymph nodes. In contrast, SD-R vaccination significantly reduced serum and lung tissue viral loads, and vaccinated piglets did not show any clinical symptoms or histopathological changes. Our results demonstrated that LNTZJ1341-2012 is a mildly virulent NADC34-like PRRSV and that the live-attenuated vaccine SD-R can prevent the onset of clinical signs upon challenge with the NADC34-like PRRSV LNTZJ1341-2012 strain, indicating that SD-R is a promising vaccine candidate for the swine industry.

## 1. Introduction

Porcine reproductive and respiratory syndrome (PRRS) is a highly destructive viral disease that affects swine globally. The disease is caused by the porcine reproductive and respiratory syndrome virus (PRRSV) and has severe economic ramifications for the pig industry worldwide [1,2]. PRRSV is an enveloped RNA virus belonging to the genus *Betaarterivirus*, family *Arteriviridae*, and order *Nidovirales*. PRRSV is divided into two species: *Betaarterivirus suid* 1 (PRRSV-1) and *Betaarterivirus suid* 2 (PRRSV-2) (ICTV2021). PRRSV has a genome length of approximately 15.3 kb and comprises at least ten open reading frames (ORFs), including ORF1a, ORF1b, ORF2a, ORF2b, ORF3-5, ORF5a, and ORF6-7 [2]. ORF1a and ORF1b encode two large replicase polyproteins, pp1a and pp1ab, that are proteolytically processed by virus-encoded proteases into at least 16 mature nonstructural proteins (Nsps) [3]. The remaining eight ORFs encode the eight structural proteins of PRRSV [4]. ORF5 encodes the major structural protein glycoprotein 5 (GP5). Due to the considerable variability of ORF5, the *GP5* gene is commonly employed for phylogenetic analyses and classification of PRRSV isolates [5].

Based on the global PRRSV classification system, PRRSV-1 is divided into three or four subtypes (subtype 1 (subtype 1 (Global)), subtype 2 (subtype Ⅰ (Russia) and subtype II), and subtype 3 (subtype Ⅲ)); PRRSV-2 is divided into nine lineages (1–9) with several sublineages [6,7,8,9]. Since PRRSV emerged in 1996, PRRSV-2 strains have been the predominant strains circulating in farms across China [10]. Before 2017, almost all of the PRRSV-2 strains in China could be classified as NADC30-like (sublineage 1.8), JXA1-like/CH-1a-like (sublineage 8.7), QYYZ-like (sublineage 3.5), or VR2332-like (sublineage 5.1) strains [11]. In 2017, the RFLP 1-7-4 (NADC34-like) strain, which had a 100-amino-acid deletion in the Nsp2 region that was identical to a deletion in the American IA/2014/NADC34 strain, was first reported in China [12,13]. The distribution of the NADC34-like (sublineage 1.5) strain began to expand gradually in 2019 [14,15,16], and in 2021, it became one of the primary circulating strains in some parts of China [17,18,19,20]. The NADC34-like strains circulating in China and the United States are characterized by complex recombination of the viral genome and large differences in pathogenicity [13,21,22,23,24,25,26].

The use of vaccines to control PRRSV has a long history. At present, a total of nine PRRSV vaccines are licensed and available in China, including one killed-virus (KV) vaccine (CH-1a) and eight modified-live-virus (MLV) vaccines (Ingelvac PRRS MLV/RespPRRS MLV, CH-1R, JXA1-P80, HuN4-F112, TJM-F92, R98, GDr180, and PC) [27]. The KV vaccine has limited effectiveness [28], and MLV vaccines are generally considered to provide complete protection against homologous strains [29], but the general view is that PRRSV MLV vaccines offer very limited protection against heterologous strains [30,31]. The MLV vaccines currently used to prevent and control PRRSV in China all belong to lineage 5 and lineage 8. At present, lineage 1 (NADC30-like, sublineage 1.8; and NADC34-like, sublineage 1.5) strains have come to represent the largest branch of PRRSV in China, and in some areas of the country, positivity for lineage 1 strains among samples can reach 64% [17]. However, existing vaccines do not provide full protection against lineage 1 strains of PRRSV [22,32,33,34,35,36,37].

In our prior research, we developed an attenuated lineage 1 PRRSV vaccine (SD-R GenBank accession: ON254650.1) that offers reliable and efficient defense against both homologous NADC30-like PRRSV and heterologous NADC30-like PRRSV challenges, ensuring safety and effectiveness [27]. SD-R can also provide protection against the lineage 8 strain HuN4 (GenBank accession: EF635006.1). Whether SD-R vaccine candidates can provide protection against NADC34-like strains remains to be investigated.

## 2. Materials and Methods

### 2.1. Sample Collection and Virus Isolation

In December 2020, pigs on a farm in Liaoning Province, China, experienced NADC34-like PRRSV infection, extensive miscarriage among sows, and the death of some piglets. Lung tissue samples were collected from dead piglets. The tissues were homogenized, the supernatant of the homogenate was used for viral detection by RT–PCR, the isolate was named LNTZJ1341-2012, and eight pairs of specific primers were used to determine the whole genome sequence of the virus [14]. RNA extraction, reverse transcription, primer sequence design, PCR procedures, and whole-genome sequencing were carried out in accordance with methods described in earlier studies [38]. The remaining suspensions were passed through 0.22 μm filters and inoculated onto primary alveolar macrophages (PAMs) and Marc-145 cells (an African green monkey kidney cell line) for virus isolation [39]. After three days, the cultures were harvested and preserved as viral stocks at a temperature of −80 °C. Cultures from the third passage in PAMs were utilized for animal experiments. The viral titer was evaluated using previously described methods [39].

### 2.2. Sequencing Analysis

All available sublineage 1.5 and sublineage 1.8 whole-genome sequences of PRRSV-2 collected from 1991 to 2022 were downloaded from GenBank and aligned with the sequences obtained in this study using MAFTT [40]. A phylogenetic tree was inferred using the neighbor-joining method and the Kimura 2-parameter substitution model (MEGA 7.0). The topology of the trees was confirmed with 1000 bootstrap replication steps [41]. Evolview (version 2.0) was used to annotate and modify the trees [42]. Deduced amino acid sequences were aligned by ClustalW in the Lasergene software (Version7.1, DNASTAR Inc., Madison, WI, USA). We utilized the Recombination Detection Program 4 (RDP4) to screen for potential recombination events in the multiple alignment of the genomes and test the role of recombination in the generation of LNTZJ1341-2012 (GenBank accession: OL516360.1) [11]. Finally, the graphics of recombination events were drawn by SimPlot v3.5.1 within a 500 bp window sliding along the genome alignment (20 bp step size).

### 2.3. Immunofluorescence Assay (IFA)

To prepare viral antigens, HP-PRRSV HUN4 (GenBank accession: EF635006.1) and NADC34-like PRRSV LNTZJ1341-2012 (GenBank accession: OL516360.1) strains were inoculated into PAMs and Marc-145 cells. Identification of the viruses was carried out through IFA using a monoclonal antibody (1H4) targeting the N protein of PRRSV-2 [43]. IFAs were performed following the methods described previously, and DAPI was used to stain the nucleus [21].

### 2.4. Animals and Experimental Design

A total of thirteen 28-day-old piglets were obtained from a commercial pig herd in Harbin with PRRSV-, ASFV-, PCV2-, PRV-, and CSFV-free status. The piglets were blindly divided into three groups: 5 piglets in group A were used for SD-R immunization and LNTZJ1341-2012 inoculation; 5 piglets in group B were used for LNTZJ1341-2012 inoculation; and 3 piglets in group C were used as negative controls (Table 1). To ensure separation, each group of piglets was housed in an individual room. Piglets belonging to group A were immunized intramuscularly with a 2 mL dose of SD-R (dose: 10^6.2^ TCID_50_/mL).

At 28 days post-vaccination (dpv), the piglets in groups A and B were infected intramuscularly (2 mL) and intranasally (2 mL), respectively, with third-passage PAMs LNTZJ1341-2012 (1 × 10^5.0^ TCID_50_/mL). The three piglets in group C were intramuscularly (2 mL) and intranasally (2 mL) administered Dulbecco’s modified Eagle’s medium (DMEM) (Table 1). The rectal body temperatures and clinical signs of the piglets were recorded daily throughout the experiment, and body weight was measured every week.

### 2.5. Serological Testing

A commercial ELISA kit from IDEXX, Inc. (Westbrook, ME, USA) was employed to measure PRRSV-specific antibodies, following the manufacturer’s instructions. The PRRSV-specific antibody titer was determined by calculating the sample-to-positive (S/P) ratio, and serum samples were classified as positive if the S/P ratio was ≥0.4.

To conduct serum neutralization (SN) assays, all sera collected at 28 dpv and 21 dpi were subjected to heat inactivation at 56 °C for 30 min. Subsequently, serial two-fold dilutions of each serum sample were prepared using DMEM as the diluent. Suspensions containing 100 TCID50 PRRSV per 100 µL were then prepared, and 100 µL aliquots were added to each serum dilution. The serum–viral mixtures were incubated for 1 h in a 37 °C water bath. Next, the mixtures were dispensed onto Marc-145 cells in 96-well plates. The plates were further incubated at 37 °C in a humidified atmosphere with 5% CO_2_ for 7 days. Duplicate samples were analyzed to determine the cytopathic effect (CPE) every day after inoculation. The neutralization titer of serum was calculated by the Reed–Muench method. A titer of ≥1:4 was considered positive. Three independent tests were performed for each serum sample.

### 2.6. Macroscopic and Histopathological Lesions

At 21 days post-infection (dpi), all piglets were humanely euthanized for pathological examination. Lung and submaxillary lymph node samples were collected from each piglet and fixed in a 4% formaldehyde solution for subsequent histopathological analysis using hematoxylin and eosin staining.

### 2.7. Assessment of Viremia and Viral Loads in Tissues

Blood was collected at 0, 7, 14, 21, and 28 dpv and 3, 5, 7, 10, 14, 17, and 21 dpi for viremia detection via real-time quantitative PCR (qPCR). qPCR was performed according to an earlier study [21]. Tissue samples were obtained from the heart, liver, spleen, lung, kidney, submaxillary lymph node, tonsil, small intestine, bladder, and stomach for viral detection by qPCR.

### 2.8. Statistical Analysis

Significant differences between two groups were determined using the *t* test (and nonparametric tests) in GraphPad 5.0 (San Diego, CA, USA). The level of significance was set at *p* < 0.05.

## 3. Results

### 3.1. Genomic Characteristics of the Vaccine Candidate Strain SD-R and NADC34-like PRRSV LNTZJ1341-2012

**Figure 1:**
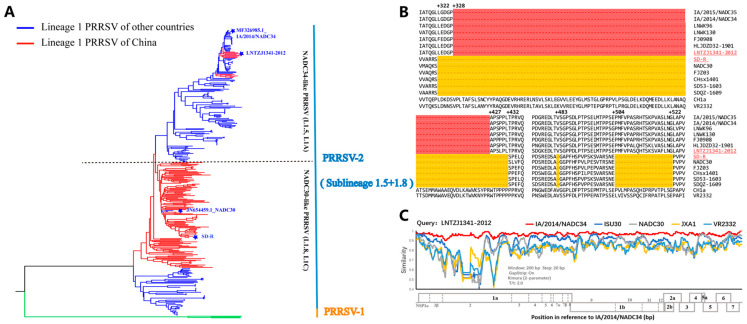
Phylogenetic analysis, recombination analysis, and deduced amino acid alignment of Nsp2 from the SD-R and LNTZJ1341-2012 strains. (**A**) Phylogenetic tree of SD-R and LNTZJ1341-2012 based on all the whole-genome sequences of sublineage 1.5 and sublineage 1.8 PRRSV strains. Red branches represent Chinese strains, and blue branches represent strains from other countries. The green branch represents PRRSV-1 strain. (**B**) The NSP2 deletion pattern in SD-R and LNTZJ1341-2012: 131 aa discontinuous deletions are labeled yellow; 100 aa continuous deletions are labeled red. (**C**) Characterization of the recombination events between representative PRRSVs of each lineage and the LNTZJ1341-2012 isolate.

In recent years, lineage 1 (sublineage 1.5 and sublineage 1.8) PRRSV-2 has become the main epidemic strain in China. To understand the genetic relationship between the SD-R and LNTZJ1341-2012 strains, we downloaded all whole-genome sequences of sublineage 1.5 and sublineage 1.8 PRRSV from NCBI for phylogenetic analysis. The results showed that SD-R and LNTZJ1341-2012 belonged to sublineage 1.8 (NADC30-like/L1C) and sublineage 1.5 (NADC34-like/L1A), respectively (Figure 1A). Although both strains belong to lineage 1, they are distantly related, with 86.9% ORF5 gene and 84.4% genome-wide nucleotide homology, respectively (Table 2). The nucleotide and amino acid similarities among different genes between SD-R and LNTZJ1341-2012 were 76.5–93.2% and 71.6–98.0%, respectively (Table 2). LNTZJ1341-2012 shared the highest homology with IA/2014/NADC34 compared with the other representative NADC34-like (sublineage 1.5/L1A) strains (IA/2014/NADC34, HLJDZD32-1901, PRRSV-ZDXYL-China-2018-1, and JS2021NADC34) (Table 2). The deduced amino acid alignment of Nsp2 showed that SD-R has a 131 aa (131 + 1 + 19) discontinuous deletion and that LNTZJ1341-2012 has a 100 aa continuous deletion (Figure 1B). Recombination analysis showed that the LNTZJ1341-2012 strain did not recombine with other strains (Figure 1C).

### 3.2. Virus Isolation and Identification

**Figure 2:**
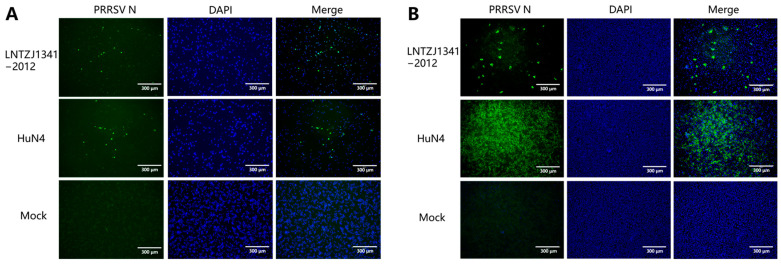
IFA images showing the reactivity of a monoclonal antibody against the PRRSV N protein to LNTZJ1341-2012-infected PAMs (**A**) and Marc-145 (**B**) cells. The nuclei were stained with DAPI. Scale bar = 300 μm.

To evaluate the pathogenicity of the newly emerging NADC34-like LNTZJ1341-2012 strain and the protective effect of the vaccine candidate strain SD-R against it, tissue homogenates of samples positive for the LNTZJ1341-2012 strain were added to PAMs and Marc-145 cells. PRRSV N-protein expression was observed in PAMs and Marc-145 cells that were inoculated with the LNTZJ1341-2012 strain during IFA testing. These findings suggested that the virus isolates replicated successfully in both PAMs and Marc-145 cells (Figure 2).

### 3.3. Clinical Reactions after Immunization and Challenge

**Figure 3:**
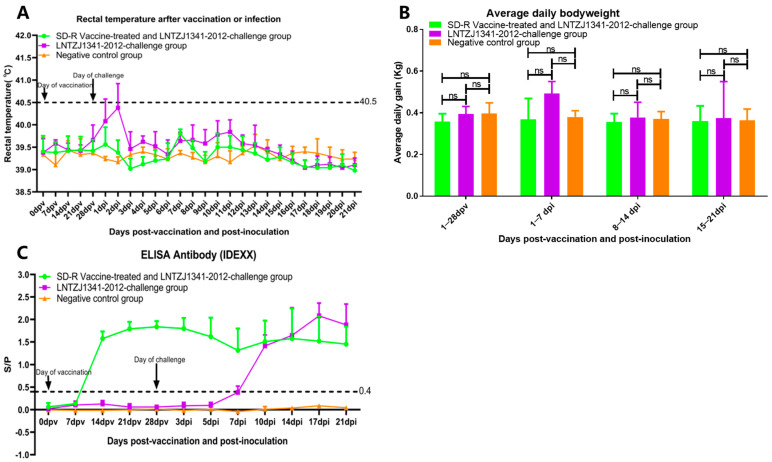
Rectal temperatures, average daily body weights, and PRRSV-specific antibody levels in the experimental piglets. (**A**) Rectal temperatures of group A, group B, and group C piglets. A rectal temperature of ≥40.5 °C was used to define fever. The means ± SDs (error bars) of the temperatures are shown. (**B**) Average daily weight gain measurements. The means ± SDs (error bars) of the body weight gain are shown; ns: no significant difference. (**C**) PRRSV-specific antibody levels were measured using an IDEXX ELISA kit. The antibody level is expressed as the S/P ratio; serum was confirmed to be positive when the S/P ratio was >0.4. The means ± SDs (error bars) of the specific antibody levels are shown.

After immunization with SD-R, no piglets in group A showed any clinical signs of PRRS, in contrast with those in group B and group C. After the LNTZJ1341-2012 challenge, some piglets in group B (3/5) showed the typical clinical symptoms of PRRSV infection, such as loss of appetite, lethargy, sleepiness, and cough. Two pigs in group B had only a transient fever (≥40.5 °C), showing fever symptoms up to 41.2 °C at 1 dpi (1/5) and 2 dpi (2/5), and then their body temperature returned to the normal range at 3 dpi, which was maintained until the end of the experiment (Figure 3A). Piglets in group A and group C did not display any clinical symptoms throughout the entire experiment. Weekly measurements of the body weight of the piglets were taken, and the results showed that there was no significant difference in daily weight gain among group A, group B, and group C (Figure 3B). Every piglet in the three groups survived until the end of the experiment without any fatalities.

### 3.4. Antibody Responses in Immunized or Challenged Piglets

PRRSV-specific antibodies were measured using IDEXX ELISA, which showed that all the immunized piglets in group A seroconverted at 14 dpv (Figure 3C). In group B, a total of two out of five piglets seroconverted by 7 dpi, and the remaining piglets seroconverted by 10 dpi (Figure 3C). During the experiment, piglets from group C showed negative serological results (Figure 3C).

### 3.5. Macroscopic and Histopathological Lesions

**Figure 4:**
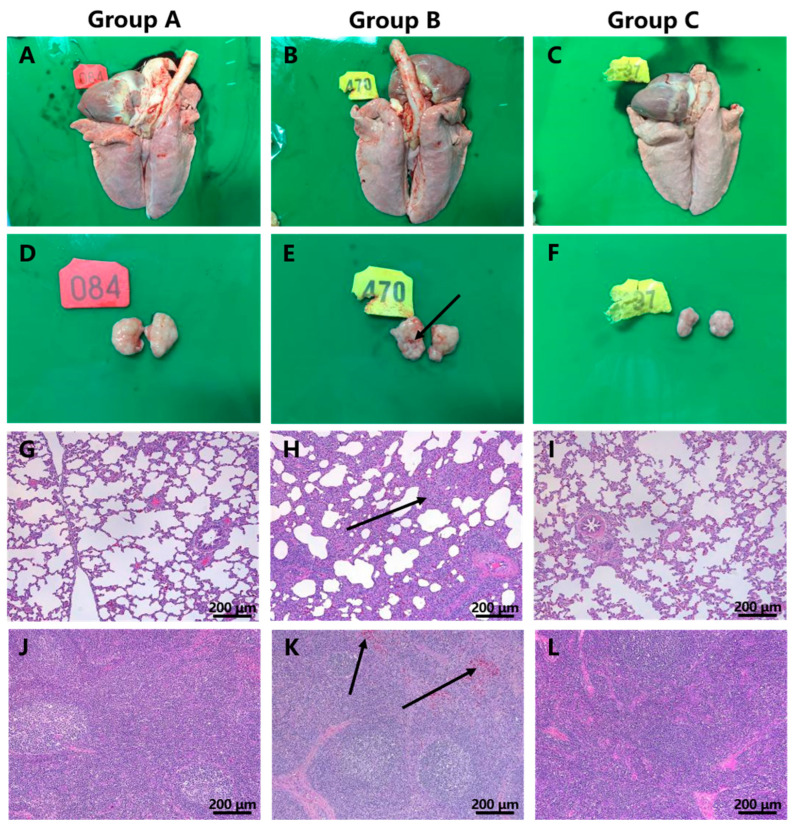
Gross and histological lesions of the lungs and submaxillary lymph nodes from group A, group B, and group C. The lungs in group B (**B**) showed no obvious lesions compared with those in groups Aand C (**A**,**C**). Hemorrhage in the submaxillary lymph nodes in group B (**E**) was observed, in contrast with groups A and C (**D**,**F**). In contrast to those in groups A and C (**G**,**I**), extensive inflammatory cell infiltration with alveolar epithelial cell proliferation and moderate alveolar diaphragm widening in the lungs were observed in group B (**H**). In contrast to that in groups A and C (**J**,**L**), scattered bleeding in the paracortical region of the submaxillary lymph nodes was observed in group B (**K**). Scale bar = 200 μm. The location of the lesion is indicated by an arrow.

All piglets were euthanized at 21 dpi (after the LNTZJ1341-2012 challenge). In contrast to the piglets in groups A and C, some piglets in group B showed lesions typical of PRRS, such as hemorrhaging in the submaxillary lymph nodes (2/5) (Figure 4B,E). Histopathology revealed extensive inflammatory cell infiltration with alveolar epithelial cell proliferation and moderate alveolar diaphragm widening in the lungs (5/5) (Figure 4H) and scattered bleeding in the paracortical region of the submaxillary lymph nodes (5/5) (Figure 4K) in group B, in contrast with that in pigs in groups A and C. No macroscopic or histopathological changes were observed in piglets in groups A and C.

### 3.6. Viremia and Viral Tissue Distribution

**Figure 5:**
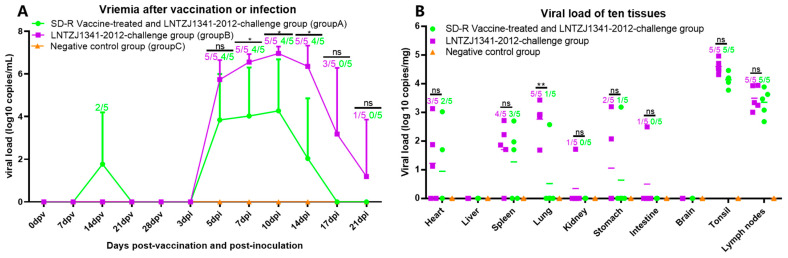
Viral load and distribution in tissues. (**A**) Serum viral load on different collection dates. (**B**) Viral loads in different tissues. The PRRSV viral load in tissues and serum from each group was determined using qPCR. Tissue samples were collected at 21 dpi, while serum was collected at 0, 7, 14, 21, and 28 dpv and at 3, 5, 7, 10, 14, 17, and 21 dpi. The data are presented as means ± SDs (error bars). Groups A, B, and C are labeled green, purple, and orange, respectively. An asterisk (*) indicates a significant difference between the groups (ns: no significant difference; *: *p* < 0.05; **: *p* < 0.01). The numbers represent the number of piglets with detectable viral loads and the number of piglets in each group.

The viral load in serum samples from individual piglets was estimated at 0, 7, 14, 21, and 28 dpv and at 3, 5, 7, 10, 14, 17, and 21 dpi. The results showed that only two of the five piglets in group A had low levels of viremia at 14 dpv (Figure 5A). From 0 to 10 dpi, piglets infected with LNTZJ1341-2012 (groups A and B) showed a gradual increase in viral load, reaching a peak at 10 dpi. Subsequently, the viral load gradually decreased. No viremia was observed in group C (Figure 5A). One piglet in group B still had detectable viremia at 21 dpi. The average viral loads at 7~14 dpi in group A were significantly lower than those in group B (Figure 5A). Moreover, the number of piglets with viremia in group A was also less than that in group B.

The hearts, livers, spleens, lungs, kidneys, stomachs, small intestines, brains, submaxillary lymph nodes, and tonsils were collected from individual piglets and analyzed by qPCR. The viral load was found to differ in different tissues in groups A and B (Figure 5B). The highest viral load was detected in the tonsils, followed by the submaxillary lymph nodes and the lungs. The average viral loads of ten tissues in group A were lower than those in group B. Notably, group A pigs had significantly lower average viral loads in their lung tissue than group B pigs (Figure 5B). Throughout the study, no viremia or viral loads were detected in the samples from group C. The samples were confirmed to contain the original virus by sequencing a portion of the ORF7 gene.

### 3.7. Serum-Neutralizing Antibody Detection

To evaluate the protective immune responses induced by SD-R immunization, SN Abs against the SD-R and LNTZJ1341-2012 strains were measured. All control wells (100 TCID50) displayed CPE on day 3 of cell inoculation. On day 7 of cell inoculation, no further changes were observed in the experimental wells. Therefore, this study chose to present the results from the 3- and 7-day post-inoculation of cells. The homologous SN Ab could be detected beginning at 28 dpv in group A, and all pigs showed higher titers of neutralizing antibodies against SD-R at 21 dpi (Table 3). In the SN assay against the LNTZJ1341-2012 strain, at 28 dpv, neither group A nor group B developed SN Abs against the LNTZJ1341-2012 strain either (Table 4). Interestingly, serum from group B pigs at 21 dpi could delay the appearance of CPE on the third day of the SN assay, and two pigs were able to completely neutralize the LNTZJ1341-2012 strain on day 7 (Table 4). In contrast, higher neutralization titers were detected in group A pigs both on day 3 and day 7 of cell inoculation (Table 4).

## 4. Discussion

NADC34-like PRRSV was first reported in the United States and has had a severe impact on the American pig industry, causing a phenomenon described as an “abortive storm” [44]. American researchers found that the enhancement of the pathogenicity of NADC34-like PRRSV was very obvious, and the IA/2014/NADC34 prototype was highly pathogenic to piglets [13]. There have been many reports on the pathogenicity of NADC34-like PRRSV in China, indicating the mild pathogenicity of the HLJDZD32-1901 strain [21], the moderate pathogenicity of the PRRSV-ZDXYL-China-2018-1 strain [26], and the high pathogenicity of the JS2021NADC34 strain [25]. In contrast to those reports, only some of the pigs challenged with the LNTZJ1341-2012 strain (group B) showed clinical symptoms typical of PRRSV (transient fever, cough, etc.), slight histopathological changes, and no significant difference in weight gain (Table 3). Therefore, the LNTZJ1341-2012 strain exhibited mild pathogenicity in piglets.

Interestingly, LNTZJ1341-2012 shared high genomic similarity with the strains HLJDZD32-1901 (95.4%), PRRSV-ZDXYL-China-2018-1 (95.1%), and JS2021NADC34 (94.1%), without any recombination with other PRRSV strains, and shared the same pattern of deletion in Nsp2 (100 aa continuous deletion). However, the pathogenicity of these viruses varies greatly, with the JS2021NADC34 strain causing a fatality rate of 75%, while the HLJDZD32-1901 and LNTZJ1341-2012 strains cause only mild clinical symptoms (Table 3). A high viral load is one of the most typical findings for highly pathogenic strains [45,46]. The LNTZJ1341-2012 strain can generate a higher serum viral load compared to the IA/2014/NADC34 strain, induce a longer duration of viremia than the JS2021NADC34 strain, and have the lowest pathogenicity (Table 5). Studies have found that there is no positive correlation between viremia and the pathogenicity of NADC34-like PRRSV strains prevalent in the United States [13], so the use of viremia to determine the pathogenicity of NADC34-like PRRSV strains does not seem to be applicable.

There are two sublineages of lineage 1 PRRSV-2 circulating in China, including NADC30-like (sublineage 1.8) and NADC34-like (sublineage 1.5) strains. These strains were first detected in China in 2012 and 2017, respectively [12,47]. Lineage 1 PRRSV-2 has become the most prevalent subtype of PRRSV strain in China [17]. Since the first PRRSV vaccine was released in China in 2005, China has used vaccines to prevent and control PRRSV. At present, many experiments have proven that vaccines, such as Ingelvac PRRS MLV/RespPRRS MLV [33,36], CH-1a [34], JXA1-P80 [36], TJM-F92 [35], HuN4-F112 [35], GDr180 [35], and R98 [37], available in the Chinese market, can provide only partial protection against NADC30-like (sublineage 1.8) strains. In a previous study, we created an attenuated lineage 1 PRRSV vaccine (SD-R) that has been proven to offer full protection against both homologous and heterologous NADC30-like PRRSV challenges [27].

To our knowledge, only one vaccine (Ingelvac PRRS MLV) has been evaluated for protection against NADC34-like PRRSV, and it provides incomplete protection against the PRRSV/CN/FJGD01/2021 strain [22]. In this study, the vaccine candidate SD-R significantly reduced the average serum viral load from 7 dpi to 14 dpi and prevented fever in the vaccinated challenged group (group A); at the same time, vaccinated piglets showed significantly lower levels of the virus in their lungs than unvaccinated piglets (group B) on the 21st day post-infection.

Previous studies have indicated that serum-neutralizing antibodies (SN Abs) play a critical role in the development of protective immunity against PRRSV [2,48]. Normally, neutralizing antibodies induced by PRRSV vaccines only provide homologous neutralizing activity and show lower/zero neutralizing titers against heterogenic strains [49,50]. In this study, the neutralizing titers against the LNTZJ1341-2012 strain were undetectable in all 28 dpv sera. Notably, sera from pigs in the challenged group at 21 dpi exhibited the ability of the LNTZJ1341-2012 strain to effectively hinder the infection of Marc-145 cells. In the vaccinated challenged group, the numbers and neutralization titers of pigs that could produce neutralizing antibodies were higher than those in the challenged group. Consequently, even though neutralizing titers were not detected at 28 dpv, prophylactic immunization with the SD-R vaccine could help animals generate heterologous neutralizing antibodies in the later stages, reducing the impact of the LNTZJ1341-2012 strain on pigs. Interestingly, pig 083 in the vaccinated group exhibited higher neutralizing titers against LNTZJ1341-2012 (1:10) compared to SD-R (1:6.32) at 21 dpi, which may be caused by LNTZJ1341-2012 inducing homologous neutralizing antibody production in infected pigs at 21 dpi.

The protective effect of SD-R against the heterologous strain LNTZJ1341-2012 regarding viremia and organ viral load was not as obvious as that of the homologous strain SD [27]. However, the vaccinated challenged pigs did not show any clinical symptoms, and pathological analyses revealed that SD-R was effective in preventing the development of gross and microscopic lung lesions in the vaccinated challenged piglets. Taken together, these findings indicate that the PRRSV vaccine candidate SD-R has the ability to provide cross-protection against NADC34-like PRRSV LNTZJ1341-2012 infection. Meanwhile, only two of the five immunized piglets presented low viral loads at 14 dpv, which further demonstrated that SD-R showed higher safety.

## 5. Conclusions

In summary, our findings indicate that LNTZJ1341-2012 is a mildly virulent NADC34-like PRRSV, and the vaccine candidate SD-R prevents the onset of clinical signs against the LNTZJ1341-2012 strain. Therefore, the vaccine candidate strain SD-R can effectively prevent and control lineage 1 PRRSV-2 infection.

## Figures and Tables

**Table 1:** Group information for animal experiments.

Groups	Number of Animals	Vaccination	Challenge
Group A: SD-R vaccinated and LNTZJ1341-2012 challenged	5 (081; 082; 083; 084; 085)	2 × 10^6.2^ TCID_50_ per pig (SD-R)	4 × 10^5.0^ TCID_50_ per pig (LNTZJ1341-2012)
Group B: LNTZJ1341-2012 challenged	5 (094; 458; 470; 481; 490)	DMEM	
Group C: Negative control group	3 (097; 098; 101)		DMEM

**Table 2:** Nucleotide and amino acid sequence similarity between LNTZJ1341-2012 and the reference strains.

Amino Acids/ Nucleotides	IA/2014/NADC34 (MF326985.1)	HLJDZD32-1901 (MN648449.1)	PRRSV-ZDXYL-China-2018-1 (MK453049.1)	JS2021NADC34 (MZ820388.1)	SD-R (ON25465031)
Whole genome	/96.6	/95.4	/95.1	/94.1	/84.4
Nsp1α	97.2/97.4	94.4/96.7	96.7/95.7	96.7/95.6	93.3/88.0
Nsp1β	93.6/95.5	91.6/93.9	89.1/92.4	90.1/91.7	75.7/80.2
Nsp2	93.3/95.4	92.1/94.2	91.5/93.6	88.3/91.9	71.6/76.5
Nsp3	97.4/96.7	96.5/95.5	97.0/95.5	94.8/93.9	89.6/84.2
Nsp4	98.0/97.4	97.1/96.7	97.5/97.1	97.5/95.8	92.2/81.7
Nsp5	97.6/96.5	96.5/95.3	94.7/94.1	93.5/91.6	89.4/84.3
Nsp6	100/97.9	100/95.8	93.8/95.8	93.8/95.8	93.8/85.4
Nsp7α	98.7/96.6	98.0/93.7	97.3/95.3	96.6/92.6	93.3/81.4
Nsp7β	95.5/96.4	91.8/93.9	90.0/93.6	91.8/92.7	82.7/80.6
Nsp8	97.8/95.6	95.6/94.8	93.3/93.3	97.8/93.3	93.3/90.4
Nsp9	99.7/97.6	98.8/96.5	99.1/96.6	99.2/95.9	96.7/88.4
Nsp10	99.5/97.1	99.5/96.4	99.1/95.8	99.3/95.8	98.0/88.9
Nsp11	98.2/97.2	97.3/95.2	96.0/95.5	97.3/94.2	92.8/85.2
Nsp12	97.4/97.0	97.4/96.1	96.8/96.1	96.8/95.5	89.0/84.4
ORF2a	97.3/97.4	94.6/96.0	94.9/95.5	93.8/94.8	81.7/84.8
ORF2b	97.3/97.7	98.6/97.3	94.6/95.9	93.2/95.9	90.5/86.9
ORF3	94.9/96.9	94.1/96.2	93.7/95.8	92.5/94.6	83.9/86.4
ORF4	98.3/96.8	96.6/96.3	98.3/96.6	97.8/95.2	93.9/92.6
ORF5	95.0/95.9	94.0/94.7	92.0/94.2	92.0/92.7	87.4/86.9
ORF5a	98.1/96.8	98.1/96.2	94.2/94.9	94.2/94.9	94.1/82.8
ORF6	97.1/97.5	97.1/96.6	97.7/96.6	97.1/96.6	93.1/92.1
ORF7	98.4/97.6	96.8/96.5	96.0/96.8	94.4/95.2	94.4/92.7

**Table 3:** Serum neutralization titers of three different groups against the SD-R strain.

Group A	Serum Neutralization Titer		Group B	Serum Neutralization Titer		Group C	Serum Neutralization Titer	
	3d	7d		3d	7d		3d	7d
081 28 dpv	5.61	5	094 28 dpv	<1:4	<1:4	097 28 dpv	<1:4	<1:4
082 28 dpv	8.03	<1:4	458 28 dpv	<1:4	<1:4	098 28 dpv	<1:4	<1:4
083 28 dpv	4	<1:4	470 28 dpv	<1:4	<1:4	101 28 dpv	<1:4	<1:4
084 28 dpv	<1:4	<1:4	481 28 dpv	<1:4	<1:4	097 21 dpi	<1:4	<1:4
085 28 dpv	5.62	<1:4	490 28 dpv	<1:4	<1:4	098 21 dpi	<1:4	<1:4
081 21 dpi	10	10	094 21 dpi	<1:4	<1:4	101 21 dpi	<1:4	<1:4
082 21 dpi	22.48	12.64	458 21 dpi	<1:4	<1:4			
083 21 dpi	10	6.32	470 21 dpi	<1:4	<1:4			
084 21 dpi	8	8	481 21 dpi	<1:4	<1:4			
085 21 dpi	20	20	490 21 dpi	<1:4	<1:4			

**Table 4:** Serum neutralization titer of three different groups against the LNTZJ1341-2012 strain.

Group A	Serum Neutralization Titer		Group B	Serum Neutralization Titer		Group C	Serum Neutralization Titer	
	3d	7d		3d	7d		3d	7d
081 28 dpv	<1:4	<1:4	094 28 dpv	<1:4	<1:4	097 28 dpv	<1:4	<1:4
082 28 dpv	<1:4	<1:4	458 28 dpv	<1:4	<1:4	098 28 dpv	<1:4	<1:4
083 28 dpv	<1:4	<1:4	470 28 dpv	<1:4	<1:4	101 28 dpv	<1:4	<1:4
084 28 dpv	<1:4	<1:4	481 28 dpv	<1:4	<1:4	097 21 dpi	<1:4	<1:4
085 28 dpv	<1:4	<1:4	490 28 dpv	<1:4	<1:4	098 21 dpi	<1:4	<1:4
081 21 dpi	8	5	094 21 dpi	5.61	<1:4	101 21 dpi	<1:4	<1:4
082 21 dpi	13.56	8	458 21 dpi	5.61	<1:4			
083 21 dpi	12.64	10	470 21 dpi	8	5			
084 21 dpi	8	5.62	481 21 dpi	6.32	5			
085 21 dpi	12.64	10	490 21 dpi	5	<1:4			

**Table 5:** Comparison of the pathogenicity of different NADC34-like strains.

Infecting PRRSV Strain	Accession No.	Days Post-Inoculation (dpi)	Inoculated Dose	Parameters Evaluated	Challenge Group	Reference
LNTZJ1341-2012	OL516360	21	4 × 10^5^ TCID_50_	Clinical symptoms	Mild clinical symptoms in	This study
				Days of fever	2/5 pigs fever for 2 days	
				Pathological and histopathological lesions	Typical pathological changes in some piglets (2/5)	
				Viremia	Peaked at 10 dpi, longer than 21 days	
IA/2014/NADC34	MF326985.1	14	5 × 10^4^ TCID_50_	Clinical symptoms	Severe clinical symptoms, 2/14 pigs died by	[13]
				Days of fever	6 days	
				Pathological and histopathological lesions	Severe pathological changes	
				Viremia	Peaked at 4 dpi, longer than 14 days	
HLJDZD32-1901	MN648449.1	14	4 × 10^4^ TCID_50_	Clinical symptoms	Mild clinical symptoms	[21]
				Days of fever	No fever	
				Pathological and histopathological lesions	Mild pathological changes	
				Viremia	Peaked at 7 dpi, longer than 14 days	
PRRSV-ZDXYL-China-2018-1	MK453049.1	28	4 × 10^3^ TCID_50_	Clinical symptoms	Mild clinical symptoms for	[26]
				Days of fever	2 days	
				Pathological and histopathological lesions	Moderate pathological changes	
				Viremia	Peaked at 11 dpi, longer than 28 days	
JS2021NADC34	MZ820388.1	14	3 × 10^6^ TCID_50_	Clinical symptoms	Severe clinical symptoms, 3/4 pigs died by	[25]
				Days of fever	8 days	
				Pathological and histopathological lesions	Severe pathological changes	
				Viremia	Peaked at 7 dpi, viremia lasting for 14 days	

## Data Availability

The original contributions presented in the study are included in the article; further inquiries can be directed to the corresponding author.

## References

[1] Zhang Z., Li Z., Li H., Yang S., Ren F., Bian T., Sun L., Zhou B., Zhou L., Qu X. (2022). The economic impact of porcine reproductive and respiratory syndrome outbreak in four Chinese farms: Based on cost and revenue analysis. Front. Vet. Sci..

[2] Lunney J.K., Fang Y., Ladinig A., Chen N., Li Y., Rowland B., Renukaradhya G.J. (2016). Porcine Reproductive and Respiratory Syndrome Virus (PRRSV): Pathogenesis and Interaction with the Immune System. Annu. Rev. Anim. Biosci..

[3] Fang Y., Snijder E.J. (2010). The PRRSV replicase: Exploring the multifunctionality of an intriguing set of nonstructural proteins. Virus Res..

[4] Kappes M.A., Faaberg K.S. (2015). PRRSV structure, replication and recombination: Origin of phenotype and genotype diversity. Virology.

[5] Murtaugh M.P., Stadejek T., Abrahante J.E., Lam T.T., Leung F.C. (2010). The ever-expanding diversity of porcine reproductive and respiratory syndrome virus. Virus Res..

[6] Stadejek T., Stankevicius A., Murtaugh M.P., Oleksiewicz M.B. (2013). Molecular evolution of PRRSV in Europe: Current state of play. Vet. Microbiol..

[7] Balka G., Podgorska K., Brar M.S., Balint A., Cadar D., Celer V., Denes L., Dirbakova Z., Jedryczko A., Marton L. (2018). Genetic diversity of PRRSV 1 in Central Eastern Europe in 1994–2014: Origin and evolution of the virus in the region. Sci. Rep..

[8] Shi M., Lam T.T., Hon C.C., Hui R.K., Faaberg K.S., Wennblom T., Murtaugh M.P., Stadejek T., Leung F.C. (2010). Molecular epidemiology of PRRSV: A phylogenetic perspective. Virus Res..

[9] Shi M., Lam T.T., Hon C.C., Murtaugh M.P., Davies P.R., Hui R.K., Li J., Wong L.T., Yip C.W., Jiang J.W. (2010). Phylogeny-based evolutionary, demographical, and geographical dissection of North American type 2 porcine reproductive and respiratory syndrome viruses. J. Virol..

[10] Guo Z., Chen X.X., Li R., Qiao S., Zhang G. (2018). The prevalent status and genetic diversity of porcine reproductive and respiratory syndrome virus in China: A molecular epidemiological perspective. Virol. J..

[11] Xu H., Xiang L., Tang Y.D., Li C., Zhao J., Gong B., Sun Q., Leng C., Peng J., Wang Q. (2022). Genome-Wide Characterization of QYYZ-Like PRRSV During 2018–2021. Front. Vet. Sci..

[12] Zhang H.L., Zhang W.L., Xiang L.R., Leng C.L., Tian Z.J., Tang Y.D., Cai X.H. (2018). Emergence of novel porcine reproductive and respiratory syndrome viruses (ORF5 RFLP 1-7-4 viruses) in China. Vet. Microbiol..

[13] van Geelen A.G.M., Anderson T.K., Lager K.M., Das P.B., Otis N.J., Montiel N.A., Miller L.C., Kulshreshtha V., Buckley A.C., Brockmeier S.L. (2018). Porcine reproductive and respiratory disease virus: Evolution and recombination yields distinct ORF5 RFLP 1-7-4 viruses with individual pathogenicity. Virology.

[14] Xu H., Song S., Zhao J., Leng C., Fu J., Li C., Tang Y.D., Xiang L., Peng J., Wang Q. (2020). A potential endemic strain in China: NADC34-like porcine reproductive and respiratory syndrome virus. Transbound. Emerg. Dis..

[15] Liu J., Wei C., Lin Z., Xia W., Ma Y., Dai A., Yang X. (2019). Full genome sequence analysis of a 1-7-4-like PRRSV strain in Fujian Province, China. PeerJ.

[16] Bao H., Li X. (2021). Emergence and spread of NADC34-like PRRSV in China. Transbound. Emerg. Dis..

[17] Xu H., Li C., Li W., Zhao J., Gong B., Sun Q., Tang Y.D., Xiang L., Leng C., Peng J. (2022). characteristics of Chinese NADC34-like PRRSV during 2020–2021. Transbound. Emerg. Dis..

[18] Zhou L., Yang Y., Xia Q., Guan Z., Zhang J., Li B., Qiu Y., Liu K., Shao D., Ma Z. (2022). Genetic characterization of porcine reproductive and respiratory syndrome virus from Eastern China during 2017–2022. Front. Microbiol..

[19] Li P., Shen Y., Wang T., Li J., Li Y., Zhao Y., Liu S., Li B., Liu M., Meng F. (2022). Epidemiological survey of PRRS and genetic variation analysis of the ORF5 gene in Shandong Province, 2020–2021. Front. Vet. Sci..

[20] Zhao H.Z., Wang F.X., Han X.Y., Guo H., Liu C.Y., Hou L.N., Wang Y.X., Zheng H., Wang L., Wen Y.J. (2022). Recent advances in the study of NADC34-like porcine reproductive and respiratory syndrome virus in China. Front. Microbiol..

[21] Song S., Xu H., Zhao J., Leng C., Xiang L., Li C., Fu J., Tang Y.D., Peng J., Wang Q. (2020). Pathogenicity of NADC34-like PRRSV HLJDZD32-1901 isolated in China. Vet. Microbiol..

[22] Liu J., Liu C., Xu Y., Yang Y., Li J., Dai A., Huang C., Luo M., Wei C. (2022). Molecular Characteristics and Pathogenicity of a Novel Recombinant Porcine Reproductive and Respiratory Syndrome Virus Strain from NADC30-, NADC34-, and JXA1-Like Strains That Emerged in China. Microbiol. Spectr..

[23] Wang X., Zhang K., Mo Q., Chen G., Lv J., Huang J., Pang Y., Wang H., Liu W., Huang K. (2022). The Emergence and Pathogenesis of Recombinant Viruses Associated with NADC34-like Strains and the Predominant Circulating Strains of Porcine Reproductive and Respiratory Syndrome Virus in Southern China. Viruses.

[24] Sun Y.F., Liu Y., Yang J., Li W.Z., Yu X.X., Wang S.Y., Li L.A., Yu H. (2022). Recombination between NADC34-like and QYYZ-like strain of porcine reproductive and respiratory syndrome virus with high pathogenicity for piglets in China. Transbound. Emerg. Dis..

[25] Yuan L., Zhu Z., Fan J., Liu P., Li Y., Li Q., Sun Z., Yu X., Lee H.S., Tian K. (2022). High Pathogenicity of a Chinese NADC34-like PRRSV on Pigs. Microbiol. Spectr..

[26] Xie C.Z., Ha Z., Zhang H., Zhang Y., Xie Y.B., Zhang H., Nan F.L., Wang Z., Zhang P., Xu W. (2020). Pathogenicity of porcine reproductive and respiratory syndrome virus (ORF5 RFLP 1-7-4 viruses) in China. Transbound. Emerg. Dis..

[27] Zhang H., Xiang L., Xu H., Li C., Tang Y.D., Gong B., Zhang W., Zhao J., Song S., Peng J. (2022). Lineage 1 Porcine Reproductive and Respiratory Syndrome Virus Attenuated Live Vaccine Provides Broad Cross-Protection against Homologous and Heterologous NADC30-Like Virus Challenge in Piglets. Vaccines.

[28] Renukaradhya G.J., Meng X.J., Calvert J.G., Roof M., Lager K.M. (2015). Inactivated and subunit vaccines against porcine reproductive and respiratory syndrome: Current status and future direction. Vaccine.

[29] Wang H., Xu Y., Feng W. (2021). Porcine Reproductive and Respiratory Syndrome Virus: Immune Escape and Application of Reverse Genetics in Attenuated Live Vaccine Development. Vaccines.

[30] Chae C. (2021). Commercial PRRS Modified-Live Virus Vaccines. Vaccines.

[31] Nan Y., Wu C., Gu G., Sun W., Zhang Y.J., Zhou E.M. (2017). Improved Vaccine against PRRSV: Current Progress and Future Perspective. Front. Microbiol..

[32] Zhou L., Yang B., Xu L., Jin H., Ge X., Guo X., Han J., Yang H. (2017). Efficacy evaluation of three modified-live virus vaccines against a strain of porcine reproductive and respiratory syndrome virus NADC30-like. Vet. Microbiol..

[33] Wei C., Dai A., Fan J., Li Y., Chen A., Zhou X., Luo M., Yang X., Liu J. (2019). Efficacy of Type 2 PRRSV vaccine against challenge with the Chinese lineage 1 (NADC30-like) PRRSVs in pigs. Sci. Rep..

[34] Li C., Liu Z., Chen K., Qian J., Hu Y., Fang S., Sun Z., Zhang C., Huang L., Zhang J. (2022). Efficacy of the Synergy Between Live-Attenuated and Inactivated PRRSV Vaccines Against a NADC30-Like Strain of Porcine Reproductive and Respiratory Syndrome Virus in 4-Week Piglets. Front. Vet. Sci..

[35] Bai X., Wang Y., Xu X., Sun Z., Xiao Y., Ji G., Li Y., Tan F., Li X., Tian K. (2016). Commercial vaccines provide limited protection to NADC30-like PRRSV infection. Vaccine.

[36] Sun Y.F., Zhou L., Bian T., Tian X.X., Ren W.K., Lu C., Zhang L., Li X.L., Cui M.S., Yang H.C. (2018). Efficacy evaluation of two commercial modified-live virus vaccines against a novel recombinant type 2 porcine reproductive and respiratory syndrome virus. Vet. Microbiol..

[37] Zhang Q., Jiang P., Song Z., Lv L., Li L., Bai J. (2016). Pathogenicity and antigenicity of a novel NADC30-like strain of porcine reproductive and respiratory syndrome virus emerged in China. Vet. Microbiol..

[38] Xiang L., Xu H., Li C., Tang Y.D., An T.Q., Li Z., Liu C., Song S., Zhao J., Leng C. (2022). Long-Term Genome Monitoring Retraces the Evolution of Novel Emerging Porcine Reproductive and Respiratory Syndrome Viruses. Front. Microbiol..

[39] Zhang H., Leng C., Ding Y., Zhai H., Li Z., Xiang L., Zhang W., Liu C., Li M., Chen J. (2019). Characterization of newly emerged NADC30-like strains of porcine reproductive and respiratory syndrome virus in China. Arch. Virol..

[40] Katoh K., Standley D.M. (2013). MAFFT multiple sequence alignment software version 7: Improvements in performance and usability. Mol. Biol. Evol..

[41] Zhang W.L., Zhang H.L., Xu H., Tang Y.D., Leng C.L., Peng J.M., Wang Q., An T.Q., Cai X.H., Fan J.H. (2019). Two novel recombinant porcine reproductive and respiratory syndrome viruses belong to sublineage 3.5 originating from sublineage 3.2. Transbound. Emerg. Dis..

[42] He Z., Zhang H., Gao S., Lercher M.J., Chen W.H., Hu S. (2016). Evolview v2: An online visualization and management tool for customized and annotated phylogenetic trees. Nucleic Acids Res..

[43] Li W., Li M., Zhang H., Li C., Xu H., Gong B., Fu J., Guo Z., Peng J., Zhou G. (2022). A Novel Immunochromatographic Strip Based on Latex Microspheres for the Rapid Detection of North American-Type Porcine Reproductive and Respiratory Syndrome Virus. Front. Microbiol..

[44] Alkhamis M.A., Perez A.M., Murtaugh M.P., Wang X., Morrison R.B. (2016). Applications of Bayesian Phylodynamic Methods in a Recent U.S. Porcine Reproductive and Respiratory Syndrome Virus Outbreak. Front. Microbiol..

[45] Morgan S.B., Graham S.P., Salguero F.J., Sanchez Cordon P.J., Mokhtar H., Rebel J.M., Weesendorp E., Bodman-Smith K.B., Steinbach F., Frossard J.P. (2013). Increased pathogenicity of European porcine reproductive and respiratory syndrome virus is associated with enhanced adaptive responses and viral clearance. Vet. Microbiol..

[46] Zhou L., Kang R., Yu J., Xie B., Chen C., Li X., Xie J., Ye Y., Xiao L., Zhang J. (2018). Genetic Characterization and Pathogenicity of a Novel Recombined Porcine Reproductive and Respiratory Syndrome Virus 2 among Nadc30-Like, Jxa1-Like, and Mlv-Like Strains. Viruses.

[47] Zhou L., Wang Z., Ding Y., Ge X., Guo X., Yang H. (2015). NADC30-like Strain of Porcine Reproductive and Respiratory Syndrome Virus, China. Emerg. Infect. Dis..

[48] Loving C.L., Osorio F.A., Murtaugh M.P., Zuckermann F.A. (2015). Innate and adaptive immunity against Porcine Reproductive and Respiratory Syndrome Virus. Vet. Immunol. Immunopathol..

[49] Zhou L., Ni Y.Y., Pineyro P., Sanford B.J., Cossaboom C.M., Dryman B.A., Huang Y.W., Cao D.J., Meng X.J. (2012). DNA shuffling of the GP3 genes of porcine reproductive and respiratory syndrome virus (PRRSV) produces a chimeric virus with an improved cross-neutralizing ability against a heterologous PRRSV strain. Virology.

[50] Vu H.L., Kwon B., Yoon K.J., Laegreid W.W., Pattnaik A.K., Osorio F.A. (2011). Immune evasion of porcine reproductive and respiratory syndrome virus through glycan shielding involves both glycoprotein 5 as well as glycoprotein 3. J. Virol..

